# Tetra-μ-benzoato-bis­{[4-(pyrrolidin-1-yl)pyridine]zinc(II)}

**DOI:** 10.1107/S1600536809052714

**Published:** 2009-12-12

**Authors:** Seung Man Yu, Kyosang Koo, Pan-Gi Kim, Cheal Kim, Youngmee Kim

**Affiliations:** a Department of Fine Chemistry, and Eco-Product and Materials Education Center, Seoul National University of Technology, Seoul 139-743, Republic of Korea; b Korea Division of Forest Disaster Management, Korea Forest Research Institute, Seoul 130-712, Republic of Korea; c Department of Forest & Environment Resources, Kyungpook National University, Sangju 742-711, Republic of Korea; d Department of Chemistry and Nano Science, Ewha Womans University, Seoul 120-750, Republic of Korea

## Abstract

The central part of the title centrosymmetric dinuclear complex, [Zn_2_(C_7_H_5_O_2_)_4_(C_9_H_12_N_2_)_2_], has a paddle-wheel conformation with four benzoate ligands bridging two symmetry-related Zn^II^ ions. The distorted square-pyramidal coordination environment around the Zn^II^ ion is completed by an N atom from a 4-(pyrrolidin-1-yl)pyridine ligand. The Zn⋯Zn separation of 2.9826 (12) Å does not represent a formal direct metal–metal bond. The Zn^II^ ion is displaced by 0.381 (1) Å from the mean plane of the four basal O atoms. Two of the C atoms of the pyrrolidine ring are disordered over two sites with refined occupancies of 0.53 (2) and 0.47 (2).

## Related literature

For crystal structures containing the [Zn_2_(O_2_CPh)_4_] unit, see: Necefoglu *et al.* (2002 ▶); Zeleňák *et al.* (2004 ▶); Karmakar *et al.* (2006 ▶); Ohmura *et al.* (2005 ▶). For the crystal structures of copper(II) and zinc(II) benzoates with quinoxaline, 6-methyl­quinoline, 3-methyl­quinoline, di-2-pyridyl ketone and *trans*-1-(2-pyrid­yl)-2-(4-pyrid­yl)ethyl­ene, see: Lee *et al.* (2008 ▶); Yu *et al.* (2008 ▶, 2009 ▶); Park *et al.* (2008 ▶); Shin *et al.* (2009 ▶); Song *et al.* (2009 ▶). For transition metal ions as the major cation contributors to the inorganic composition of natural water and biological fluids, see: Daniele *et al.* (2008 ▶); Parkin (2004 ▶); Tshuva & Lippard (2004 ▶).
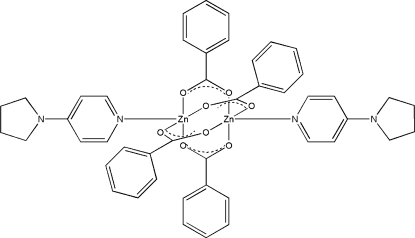


## Experimental

### 

#### Crystal data

[Zn_2_(C_7_H_5_O_2_)_4_(C_9_H_12_N_2_)_2_]
*M* *_r_* = 911.59
Monoclinic, 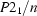

*a* = 11.0021 (11) Å
*b* = 11.4303 (11) Å
*c* = 16.9508 (16) Å
β = 93.869 (2)°
*V* = 2126.8 (4) Å^3^
*Z* = 2
Mo *K*α radiation
μ = 1.19 mm^−1^
*T* = 293 K
0.08 × 0.08 × 0.01 mm

#### Data collection

Bruker SMART CCD diffractometer
Absorption correction: multi-scan (*SADABS*; Bruker, 1997 ▶) *T*_min_ = 0.909, *T*_max_ = 0.988
11271 measured reflections
4159 independent reflections
2284 reflections with *I* > 2σ(*I*)
*R*_int_ = 0.068

#### Refinement

*R*[*F* ^2^ > 2σ(*F* ^2^)] = 0.053
*wR*(*F* ^2^) = 0.179
*S* = 0.90
4159 reflections
270 parameters
7 restraints
H-atom parameters constrained
Δρ_max_ = 0.94 e Å^−3^
Δρ_min_ = −0.71 e Å^−3^

### 

Data collection: *SMART* (Bruker, 1997 ▶); cell refinement: *SAINT* (Bruker, 1997 ▶); data reduction: *SAINT*; program(s) used to solve structure: *SHELXS97* (Sheldrick, 2008 ▶); program(s) used to refine structure: *SHELXL97* (Sheldrick, 2008 ▶); molecular graphics: *SHELXTL* (Sheldrick, 2008 ▶); software used to prepare material for publication: *SHELXTL*.

## Supplementary Material

Crystal structure: contains datablocks I, global. DOI: 10.1107/S1600536809052714/lh2962sup1.cif

Structure factors: contains datablocks I. DOI: 10.1107/S1600536809052714/lh2962Isup2.hkl

Additional supplementary materials:  crystallographic information; 3D view; checkCIF report

## Figures and Tables

**Fig. 1.**
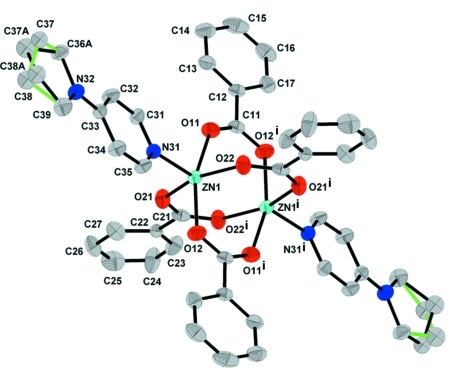
The molecular structure of the title complex showing the atom-labeling scheme. Displacement ellipsoids are shown at the 30% probability level. H atoms have been omitted for clarity. The disordered part of pyrrol group is shown by green bonds [Symmetry code: (i) -x, -y + 1, -z].

## supplementary crystallographic information

### Comment

Recently, great attention has been paid to transition metal ions as the major cation contributors to the inorganic composition of natural water and biological fluids (Daniele, *et al.*, 2008; Parkin, 2004; Tshuva & Lippard, 2004). Some biologically active molecules that have potential interactions with transition metal ions are amino acids, proteins, sugars, nucleotides, fulvic acids and humic acids. In particular, the study on the interaction of transition metal ions with fulvic acids and humic acids, mainly found in soil, is being extensively investigated. As models to examine these interactions we have previously used copper(II) and zinc(II) benzoates as building blocks and reported the structures of copper(II) and zinc(II) benzoates with quinoxaline, 6-methylquinoline, 3-methylquinoline, di-2-pyridylketone, and*trans*-1-(2-pyridyl)-2-(4-pyridyl)ethylene (Lee, *et al.*, 2008; Yu, *et al.*, 2008; Park, *et al.*, 2008; Shin, *et al.*, 2009; Yu, *et al.*, 2009; Song, *et al.*, 2009). The related paddle-wheel type structures for Zn complexes have been previouly reported (Necefoglu, *et al.*, 2002; Zeleňák, *et al.*,2004; Kamakar, *et al.*, 2006; Ohmura, *et al.*, 2005). In this work, we have employed zinc(II) benzoate as a building block and 4-(pyrrolidin-1-yl)pyridine as a ligand. We report herein the structure of the title complex.

The molecular structure of the title complex is shown in Fig. 1. The asymmetric unit contains half of the complex with the formula unit being generated by an inversion center. The central part of the complex had a paddle-wheel type conformation four benzoate ligands bridging two symmetry related Zn^II^ ions. The distorted square-pyramidal coordination environment around the unique Zn^II^ ion is completed by an N atom from a 4-(pyrrolidin-1-yl)pyridine ligand. The Zn^II^ ion is displaced by 0.381 (1) Å from the mean plane of the four basal oxygen atoms.

### Experimental

30.4 mg (0.1 mmol) of Zn(NO_3_)_2_^.^6H_2_O and 28.0 mg (0.2 mmol) of C_6_H_5_COONH_4_ were dissolved in 4 ml H_2_O and carefully layered by 4 ml me thanol solution of 4-(pyrrolidin-1-yl)pyridine (30.3 mg, 0.2 mmol). Suitable crystals of the title compound for X-ray analysis were obtained in a few weeks.

### Refinement

H atoms were placed in calculated positions with C—H distances of 0.93 Å (pyridine) and 0.97 Å (pyrrolidine). They were included in the refinement in a riding-motion approximation with Uĩso~(H) = 1.2U~eq~(C). The atoms C37/C37A and C38/C38A are disorder components both with refined occupancies of 0.53 (2) and 0.47 (2).

### Crystal data

**Table:**

[Zn_2_(C_7_H_5_O_2_)_4_(C_9_H_12_N_2_)_2_]	*F*(000) = 944
*M**_r_* = 911.59	*D*_x_ = 1.423 Mg m^−^^3^
Monoclinic, *P*2_1_/*n*	Mo *K*α radiation, λ = 0.71073 Å
Hall symbol: -P 2yn	Cell parameters from 1481 reflections
*a* = 11.0021 (11) Å	θ = 2.3–19.2°
*b* = 11.4303 (11) Å	µ = 1.19 mm^−^^1^
*c* = 16.9508 (16) Å	*T* = 293 K
β = 93.869 (2)°	Plate, colorless
*V* = 2126.8 (4) Å^3^	0.08 × 0.08 × 0.01 mm
*Z* = 2	

### Data collection

**Table:**

Bruker SMART CCD diffractometer	4159 independent reflections
Radiation source: fine-focus sealed tube	2284 reflections with *I* > 2σ(*I*)
graphite	*R*_int_ = 0.068
φ and ω scans	θ_max_ = 26.0°, θ_min_ = 2.1°
Absorption correction: multi-scan (*SADABS*; Bruker, 1997)	*h* = −13→13
*T*_min_ = 0.909, *T*_max_ = 0.988	*k* = −13→14
11271 measured reflections	*l* = −17→20

### Refinement

**Table:**

Refinement on *F*^2^	Primary atom site location: structure-invariant direct methods
Least-squares matrix: full	Secondary atom site location: difference Fourier map
*R*[*F*^2^ > 2σ(*F*^2^)] = 0.053	Hydrogen site location: inferred from neighbouring sites
*wR*(*F*^2^) = 0.179	H-atom parameters constrained
*S* = 0.90	*w* = 1/[σ^2^(*F*_o_^2^) + (0.1079*P*)^2^] where *P* = (*F*_o_^2^ + 2*F*_c_^2^)/3
4159 reflections	(Δ/σ)_max_ < 0.001
270 parameters	Δρ_max_ = 0.94 e Å^−^^3^
7 restraints	Δρ_min_ = −0.71 e Å^−^^3^

### Special details

**Table:**

Geometry. All e.s.d.'s (except the e.s.d. in the dihedral angle between two l.s. planes) are estimated using the full covariance matrix. The cell e.s.d.'s are taken into account individually in the estimation of e.s.d.'s in distances, angles and torsion angles; correlations between e.s.d.'s in cell parameters are only used when they are defined by crystal symmetry. An approximate (isotropic) treatment of cell e.s.d.'s is used for estimating e.s.d.'s involving l.s. planes.
Refinement. Refinement of *F*^2^ against ALL reflections. The weighted *R*-factor *wR* and goodness of fit *S* are based on *F*^2^, conventional *R*-factors *R* are based on *F*, with *F* set to zero for negative *F*^2^. The threshold expression of *F*^2^ > σ(*F*^2^) is used only for calculating *R*-factors(gt) *etc*. and is not relevant to the choice of reflections for refinement. *R*-factors based on *F*^2^ are statistically about twice as large as those based on *F*, and *R*- factors based on ALL data will be even larger.

### Fractional atomic coordinates and isotropic or equivalent isotropic displacement parameters (Å^2^)

**Table:**

	*x*	*y*	*z*	*U*_iso_*/*U*_eq_	Occ. (<1)
Zn1	0.11885 (5)	0.44449 (5)	0.02560 (4)	0.0380 (2)	
O11	0.1576 (4)	0.5331 (3)	−0.0760 (2)	0.0524 (11)	
O12	0.0192 (4)	0.3879 (4)	0.1160 (2)	0.0574 (11)	
O21	0.1591 (3)	0.5941 (3)	0.0872 (2)	0.0528 (10)	
O22	0.0201 (4)	0.3274 (3)	−0.0446 (2)	0.0562 (11)	
N31	0.2720 (4)	0.3488 (4)	0.0436 (2)	0.0389 (10)	
N32	0.5772 (4)	0.1339 (4)	0.0637 (3)	0.0494 (12)	
C11	0.0949 (5)	0.5977 (5)	−0.1189 (3)	0.0409 (13)	
C12	0.1545 (5)	0.6638 (4)	−0.1822 (3)	0.0428 (14)	
C13	0.2792 (6)	0.6570 (5)	−0.1882 (3)	0.0556 (16)	
H13	0.3250	0.6061	−0.1554	0.067*	
C14	0.3364 (7)	0.7242 (6)	−0.2419 (4)	0.072 (2)	
H14	0.4207	0.7224	−0.2436	0.086*	
C15	0.2653 (9)	0.7955 (7)	−0.2942 (5)	0.089 (3)	
H15	0.3025	0.8385	−0.3325	0.107*	
C16	0.1429 (8)	0.8024 (6)	−0.2898 (4)	0.074 (2)	
H16	0.0974	0.8523	−0.3236	0.089*	
C17	0.0854 (6)	0.7365 (5)	−0.2356 (3)	0.0542 (15)	
H17	0.0011	0.7397	−0.2342	0.065*	
C21	0.0822 (6)	0.6758 (5)	0.0839 (3)	0.0444 (14)	
C22	0.1148 (5)	0.7854 (4)	0.1296 (3)	0.0384 (13)	
C23	0.0408 (6)	0.8808 (5)	0.1229 (4)	0.0638 (18)	
H23	−0.0297	0.8774	0.0894	0.077*	
C24	0.0676 (7)	0.9824 (6)	0.1645 (5)	0.084 (3)	
H24	0.0174	1.0476	0.1580	0.101*	
C25	0.1670 (8)	0.9853 (6)	0.2142 (5)	0.082 (2)	
H25	0.1835	1.0527	0.2438	0.098*	
C26	0.2463 (7)	0.8915 (6)	0.2234 (4)	0.0710 (19)	
H26	0.3159	0.8950	0.2576	0.085*	
C27	0.2173 (6)	0.7926 (5)	0.1794 (4)	0.0579 (17)	
H27	0.2695	0.7286	0.1838	0.069*	
C31	0.3724 (5)	0.3727 (5)	0.0069 (3)	0.0455 (14)	
H31	0.3719	0.4391	−0.0249	0.055*	
C32	0.4768 (5)	0.3064 (5)	0.0126 (4)	0.0531 (16)	
H32	0.5441	0.3287	−0.0142	0.064*	
C33	0.4810 (5)	0.2046 (5)	0.0591 (3)	0.0422 (13)	
C34	0.3747 (5)	0.1802 (5)	0.0984 (4)	0.0517 (16)	
H34	0.3719	0.1152	0.1312	0.062*	
C35	0.2759 (5)	0.2528 (5)	0.0879 (3)	0.0478 (15)	
H35	0.2066	0.2335	0.1136	0.057*	
C36	0.6859 (5)	0.1549 (5)	0.0198 (4)	0.0573 (17)	
H36A	0.7297	0.2235	0.0398	0.069*	0.47 (2)
H36B	0.6639	0.1656	−0.0361	0.069*	0.47 (2)
C37	0.7631 (14)	0.0439 (11)	0.0341 (9)	0.054 (4)*	0.47 (2)
H37A	0.7416	−0.0159	−0.0050	0.065*	0.47 (2)
H37B	0.8495	0.0607	0.0340	0.065*	0.47 (2)
C38	0.7273 (7)	0.008 (2)	0.1161 (9)	0.067 (6)*	0.47 (2)
H38A	0.7685	0.0566	0.1567	0.080*	0.47 (2)
H38B	0.7467	−0.0731	0.1268	0.080*	0.47 (2)
C39	0.5882 (5)	0.0289 (5)	0.1122 (4)	0.0607 (18)	
H39A	0.5441	−0.0362	0.0872	0.073*	0.47 (2)
H39B	0.5595	0.0420	0.1643	0.073*	0.47 (2)
H36C	0.7113	0.2360	0.0238	0.069*	0.53 (2)
H36D	0.6710	0.1345	−0.0355	0.069*	0.53 (2)
C37A	0.7824 (9)	0.0733 (12)	0.0611 (11)	0.073 (5)*	0.53 (2)
H37C	0.8362	0.0425	0.0231	0.088*	0.53 (2)
H37D	0.8310	0.1156	0.1016	0.088*	0.53 (2)
C38A	0.7126 (9)	−0.0257 (12)	0.0979 (11)	0.063 (5)*	0.53 (2)
H38C	0.7539	−0.0514	0.1473	0.076*	0.53 (2)
H38D	0.7034	−0.0919	0.0622	0.076*	0.53 (2)
H39C	0.5231	−0.0254	0.0971	0.073*	0.53 (2)
H39D	0.5839	0.0486	0.1676	0.073*	0.53 (2)

### Atomic displacement parameters (Å^2^)

**Table:**

	*U*^11^	*U*^22^	*U*^33^	*U*^12^	*U*^13^	*U*^23^
Zn1	0.0382 (4)	0.0339 (3)	0.0421 (4)	0.0065 (3)	0.0043 (3)	0.0011 (3)
O11	0.061 (3)	0.051 (2)	0.047 (2)	0.011 (2)	0.011 (2)	0.017 (2)
O12	0.055 (3)	0.064 (3)	0.056 (3)	0.007 (2)	0.016 (2)	0.016 (2)
O21	0.052 (3)	0.040 (2)	0.064 (3)	0.007 (2)	−0.011 (2)	−0.010 (2)
O22	0.054 (3)	0.050 (2)	0.063 (3)	−0.002 (2)	−0.010 (2)	−0.014 (2)
N31	0.038 (3)	0.038 (2)	0.041 (3)	0.005 (2)	0.002 (2)	0.005 (2)
N32	0.043 (3)	0.052 (3)	0.053 (3)	0.016 (2)	0.006 (2)	0.010 (2)
C11	0.055 (4)	0.034 (3)	0.035 (3)	−0.005 (3)	0.012 (3)	−0.003 (2)
C12	0.053 (4)	0.037 (3)	0.039 (3)	−0.004 (3)	0.012 (3)	−0.004 (3)
C13	0.063 (4)	0.061 (4)	0.044 (3)	−0.008 (3)	0.013 (3)	−0.005 (3)
C14	0.073 (5)	0.076 (5)	0.068 (5)	−0.023 (4)	0.027 (4)	−0.015 (4)
C15	0.135 (8)	0.071 (5)	0.066 (5)	−0.035 (5)	0.046 (6)	0.000 (4)
C16	0.109 (7)	0.066 (5)	0.046 (4)	−0.005 (4)	0.004 (4)	0.018 (3)
C17	0.063 (4)	0.051 (4)	0.048 (4)	−0.004 (3)	−0.001 (3)	0.008 (3)
C21	0.062 (4)	0.035 (3)	0.037 (3)	−0.002 (3)	0.017 (3)	0.004 (2)
C22	0.037 (3)	0.041 (3)	0.037 (3)	−0.007 (2)	0.005 (3)	−0.005 (2)
C23	0.047 (4)	0.049 (4)	0.096 (5)	0.006 (3)	0.003 (4)	−0.018 (4)
C24	0.062 (5)	0.060 (5)	0.128 (7)	0.012 (4)	−0.026 (5)	−0.038 (5)
C25	0.103 (7)	0.052 (4)	0.093 (6)	−0.013 (4)	0.022 (5)	−0.028 (4)
C26	0.083 (5)	0.068 (5)	0.059 (4)	−0.015 (4)	−0.016 (4)	−0.013 (4)
C27	0.080 (5)	0.038 (3)	0.054 (4)	0.000 (3)	0.000 (4)	0.002 (3)
C31	0.040 (3)	0.046 (3)	0.050 (3)	0.003 (3)	0.001 (3)	0.015 (3)
C32	0.048 (4)	0.055 (4)	0.058 (4)	0.004 (3)	0.016 (3)	0.012 (3)
C33	0.042 (3)	0.041 (3)	0.044 (3)	0.009 (3)	0.001 (3)	−0.001 (3)
C34	0.053 (4)	0.043 (3)	0.061 (4)	0.011 (3)	0.012 (3)	0.017 (3)
C35	0.043 (3)	0.043 (3)	0.059 (4)	0.005 (3)	0.016 (3)	0.011 (3)
C36	0.047 (4)	0.064 (4)	0.062 (4)	0.015 (3)	0.013 (3)	0.006 (3)
C39	0.061 (4)	0.050 (4)	0.072 (4)	0.017 (3)	0.006 (4)	0.009 (3)
C36A	0.047 (4)	0.064 (4)	0.062 (4)	0.015 (3)	0.013 (3)	0.006 (3)
C39A	0.061 (4)	0.050 (4)	0.072 (4)	0.017 (3)	0.006 (4)	0.009 (3)

### Geometric parameters (Å, °)

**Table:**

Zn1—N31	2.014 (4)	C23—H23	0.9300
Zn1—O21	2.036 (4)	C24—C25	1.335 (11)
Zn1—O12	2.048 (4)	C24—H24	0.9300
Zn1—O22	2.053 (4)	C25—C26	1.384 (11)
Zn1—O11	2.068 (4)	C25—H25	0.9300
Zn1—Zn1^i^	2.9826 (12)	C26—C27	1.380 (8)
O11—C11	1.216 (6)	C26—H26	0.9300
O12—C11^i^	1.270 (6)	C27—H27	0.9300
O21—C21	1.260 (6)	C31—C32	1.374 (7)
O22—C21^i^	1.268 (7)	C31—H31	0.9300
N31—C35	1.329 (6)	C32—C33	1.404 (7)
N31—C31	1.333 (6)	C32—H32	0.9300
N32—C33	1.330 (6)	C33—C34	1.412 (7)
N32—C39	1.454 (7)	C34—C35	1.370 (7)
N32—C36	1.470 (7)	C34—H34	0.9300
C11—O12^i^	1.270 (6)	C35—H35	0.9300
C11—C12	1.500 (7)	C36—C37	1.536 (7)
C12—C13	1.384 (8)	C36—H36A	0.9700
C12—C17	1.413 (8)	C36—H36B	0.9700
C13—C14	1.375 (8)	C37—C38	1.525 (10)
C13—H13	0.9300	C37—H37A	0.9700
C14—C15	1.403 (11)	C37—H37B	0.9700
C14—H14	0.9300	C38—C39	1.545 (7)
C15—C16	1.356 (10)	C38—H38A	0.9700
C15—H15	0.9300	C38—H38B	0.9700
C16—C17	1.374 (8)	C39—H39A	0.9700
C16—H16	0.9300	C39—H39B	0.9700
C17—H17	0.9300	C37A—C38A	1.524 (10)
C21—O22^i^	1.268 (7)	C37A—H37C	0.9700
C21—C22	1.504 (7)	C37A—H37D	0.9700
C22—C23	1.361 (8)	C38A—H38C	0.9700
C22—C27	1.364 (8)	C38A—H38D	0.9700
C23—C24	1.380 (9)		
			
N31—Zn1—O21	103.14 (17)	C25—C24—H24	120.6
N31—Zn1—O12	101.53 (16)	C23—C24—H24	120.6
O21—Zn1—O12	89.46 (17)	C24—C25—C26	122.3 (7)
N31—Zn1—O22	97.95 (17)	C24—C25—H25	118.9
O21—Zn1—O22	158.90 (16)	C26—C25—H25	118.9
O12—Zn1—O22	86.50 (17)	C27—C26—C25	117.0 (7)
N31—Zn1—O11	100.06 (16)	C27—C26—H26	121.5
O21—Zn1—O11	88.04 (16)	C25—C26—H26	121.5
O12—Zn1—O11	158.27 (16)	C22—C27—C26	122.3 (6)
O22—Zn1—O11	88.11 (16)	C22—C27—H27	118.8
N31—Zn1—Zn1^i^	169.40 (13)	C26—C27—H27	118.8
O21—Zn1—Zn1^i^	87.02 (11)	N31—C31—C32	124.7 (5)
O12—Zn1—Zn1^i^	81.31 (12)	N31—C31—H31	117.7
O22—Zn1—Zn1^i^	71.90 (12)	C32—C31—H31	117.7
O11—Zn1—Zn1^i^	77.01 (12)	C31—C32—C33	119.5 (5)
C11—O11—Zn1	130.8 (4)	C31—C32—H32	120.2
C11^i^—O12—Zn1	124.6 (4)	C33—C32—H32	120.2
C21—O21—Zn1	118.5 (4)	N32—C33—C32	122.2 (5)
C21^i^—O22—Zn1	137.7 (4)	N32—C33—C34	122.2 (5)
C35—N31—C31	115.9 (4)	C32—C33—C34	115.6 (5)
C35—N31—Zn1	122.0 (3)	C35—C34—C33	119.6 (5)
C31—N31—Zn1	122.0 (4)	C35—C34—H34	120.2
C33—N32—C39	124.8 (4)	C33—C34—H34	120.2
C33—N32—C36	122.8 (5)	N31—C35—C34	124.7 (5)
C39—N32—C36	112.4 (4)	N31—C35—H35	117.6
O11—C11—O12^i^	125.3 (5)	C34—C35—H35	117.6
O11—C11—C12	118.4 (5)	N32—C36—C37	104.2 (6)
O12^i^—C11—C12	116.3 (5)	N32—C36—H36A	110.9
C13—C12—C17	118.6 (5)	C37—C36—H36A	110.9
C13—C12—C11	120.5 (6)	N32—C36—H36B	110.9
C17—C12—C11	120.8 (5)	C37—C36—H36B	110.9
C14—C13—C12	121.2 (7)	H36A—C36—H36B	108.9
C14—C13—H13	119.4	C38—C37—C36	100.9 (9)
C12—C13—H13	119.4	C38—C37—H37A	111.6
C13—C14—C15	118.8 (7)	C36—C37—H37A	111.6
C13—C14—H14	120.6	C38—C37—H37B	111.6
C15—C14—H14	120.6	C36—C37—H37B	111.6
C16—C15—C14	120.8 (6)	H37A—C37—H37B	109.4
C16—C15—H15	119.6	C37—C38—C39	103.7 (8)
C14—C15—H15	119.6	C37—C38—H38A	111.0
C15—C16—C17	120.6 (7)	C39—C38—H38A	111.0
C15—C16—H16	119.7	C37—C38—H38B	111.0
C17—C16—H16	119.7	C39—C38—H38B	111.0
C16—C17—C12	119.9 (6)	H38A—C38—H38B	109.0
C16—C17—H17	120.0	N32—C39—C38	101.2 (8)
C12—C17—H17	120.0	N32—C39—H39A	111.5
O21—C21—O22^i^	124.9 (5)	C38—C39—H39A	111.5
O21—C21—C22	117.3 (6)	N32—C39—H39B	111.5
O22^i^—C21—C22	117.9 (5)	C38—C39—H39B	111.5
C23—C22—C27	118.0 (5)	H39A—C39—H39B	109.3
C23—C22—C21	120.2 (5)	C38A—C37A—H37C	110.4
C27—C22—C21	121.8 (5)	C38A—C37A—H37D	110.4
C22—C23—C24	121.6 (7)	H37C—C37A—H37D	108.6
C22—C23—H23	119.2	C37A—C38A—H38C	111.0
C24—C23—H23	119.2	C37A—C38A—H38D	111.0
C25—C24—C23	118.8 (7)	H38C—C38A—H38D	109.0
			
N31—Zn1—O11—C11	173.5 (5)	C14—C15—C16—C17	−2.3 (11)
O21—Zn1—O11—C11	−83.5 (5)	C15—C16—C17—C12	2.2 (10)
O12—Zn1—O11—C11	0.1 (8)	C13—C12—C17—C16	−2.8 (9)
O22—Zn1—O11—C11	75.8 (5)	C11—C12—C17—C16	175.6 (5)
Zn1^i^—Zn1—O11—C11	3.9 (5)	Zn1—O21—C21—O22^i^	0.0 (7)
N31—Zn1—O12—C11^i^	−162.7 (4)	Zn1—O21—C21—C22	−179.7 (3)
O21—Zn1—O12—C11^i^	94.0 (5)	O21—C21—C22—C23	174.1 (5)
O22—Zn1—O12—C11^i^	−65.2 (5)	O22^i^—C21—C22—C23	−5.6 (7)
O11—Zn1—O12—C11^i^	10.7 (8)	O21—C21—C22—C27	−6.7 (7)
Zn1^i^—Zn1—O12—C11^i^	7.0 (4)	O22^i^—C21—C22—C27	173.6 (5)
N31—Zn1—O21—C21	176.1 (4)	C27—C22—C23—C24	0.3 (9)
O12—Zn1—O21—C21	−82.2 (4)	C21—C22—C23—C24	179.5 (6)
O22—Zn1—O21—C21	−3.3 (7)	C22—C23—C24—C25	−2.0 (12)
O11—Zn1—O21—C21	76.2 (4)	C23—C24—C25—C26	2.4 (13)
Zn1^i^—Zn1—O21—C21	−0.8 (4)	C24—C25—C26—C27	−1.0 (12)
N31—Zn1—O22—C21^i^	−175.1 (5)	C23—C22—C27—C26	1.1 (9)
O21—Zn1—O22—C21^i^	4.4 (8)	C21—C22—C27—C26	−178.1 (5)
O12—Zn1—O22—C21^i^	83.8 (5)	C25—C26—C27—C22	−0.8 (10)
O11—Zn1—O22—C21^i^	−75.2 (5)	C35—N31—C31—C32	−0.5 (9)
Zn1^i^—Zn1—O22—C21^i^	1.8 (5)	Zn1—N31—C31—C32	−175.5 (5)
O21—Zn1—N31—C35	110.2 (4)	N31—C31—C32—C33	0.8 (10)
O12—Zn1—N31—C35	18.1 (5)	C39—N32—C33—C32	178.5 (6)
O22—Zn1—N31—C35	−70.0 (4)	C36—N32—C33—C32	−1.1 (9)
O11—Zn1—N31—C35	−159.4 (4)	C39—N32—C33—C34	−3.7 (9)
Zn1^i^—Zn1—N31—C35	−86.5 (8)	C36—N32—C33—C34	176.7 (6)
O21—Zn1—N31—C31	−75.2 (5)	C31—C32—C33—N32	176.6 (6)
O12—Zn1—N31—C31	−167.3 (4)	C31—C32—C33—C34	−1.3 (9)
O22—Zn1—N31—C31	104.7 (4)	N32—C33—C34—C35	−176.4 (6)
O11—Zn1—N31—C31	15.2 (5)	C32—C33—C34—C35	1.6 (9)
Zn1^i^—Zn1—N31—C31	88.1 (8)	C31—N31—C35—C34	0.8 (9)
Zn1—O11—C11—O12^i^	−10.9 (9)	Zn1—N31—C35—C34	175.8 (5)
Zn1—O11—C11—C12	171.0 (3)	C33—C34—C35—N31	−1.4 (10)
O11—C11—C12—C13	−4.1 (8)	C33—N32—C36—C37	−172.3 (9)
O12^i^—C11—C12—C13	177.6 (5)	C39—N32—C36—C37	8.1 (10)
O11—C11—C12—C17	177.5 (5)	N32—C36—C37—C38	−30.5 (16)
O12^i^—C11—C12—C17	−0.7 (7)	C36—C37—C38—C39	42 (2)
C17—C12—C13—C14	3.6 (9)	C33—N32—C39—C38	−162.0 (9)
C11—C12—C13—C14	−174.8 (5)	C36—N32—C39—C38	17.6 (10)
C12—C13—C14—C15	−3.6 (9)	C37—C38—C39—N32	−36.8 (17)
C13—C14—C15—C16	3.0 (11)		

Symmetry codes: (i) −*x*, −*y*+1, −*z*.
